# Long-term safety and effectiveness of hybrid coronary revascularization compared to conventional revascularization strategies: a systematic review and meta-analysis

**DOI:** 10.1097/MS9.0000000000004561

**Published:** 2025-12-11

**Authors:** Singam Shashank, Rashmikaa Netyam, Chiranjeevee R. Saravanan, Nikhil D. Kolanu, Nanditha Karra, Harshada S. Keluskar, Ziad Helaly, Mohammed H. Albitar, Abdullah Sharbek, Seba Albitar, Pugazhendi Inban, Mohammed D.M. Marsool, Omniat A. Hussin

**Affiliations:** a General Medicine, Hampshire Hospitals, NHS Foundation Trust, Basingstoke, UK; b Internal Medicine, Madras Medical College, India; c Internal Medicine, China Medical University, Shenyang, China; d Internal Medicine, Osmania Medical College, Hyderabad, India; e Internal Medicine, Kazan State Medical University, Russia; f College of Medicine, Alfaisal University, Riyadh, Saudi Arabia; g Internal Medicine, St. Mary’s General Hospital and Saint Clare’s Health, New York; h Internal Medicine, Mayo Clinic, Scottsdale, Phoenix, Arizona; i Internal Medicine, Manhal University, Khartoum, Sudan

**Keywords:** coronary artery bypass, hybrid procedures, multivessel coronary disease, myocardial revascularization, percutaneous coronary intervention

## Abstract

**Background:**

Hybrid coronary revascularization (HCR), a combination of percutaneous coronary intervention and coronary artery bypass grafting (CABG), is an emerging treatment for patients with multivessel coronary artery disease (MVCAD). While traditional CABG is considered the standard, HCR may mitigate issues like saphenous vein graft failure.

**Methods:**

We systematically searched PubMed, Scopus, Web of Science, and Embase until September 2024, targeting randomized controlled trials (RCTs) that evaluated HCR against CABG in MVCAD patients. The quality of included studies was assessed using the ROB2 tool, and data were analyzed using RevMan 5.4. Four RCTs (*n* = 382) involving 382 participants were analyzed using a random-effects model. Risk of bias was assessed using the ROB2 tool.

**Results:**

Four RCTs (*n* = 382) met inclusion criteria. At 30 days, there were no statistically significant differences between HCR and CABG in all-cause death (risk ratios [RR] = 1.35, *P* = 0.74), stroke (RR = 0.99, *P* = 0.99), myocardial infarction (RR = 1.17, *P* = 0.74), or blood transfusion (RR = 0.70, *P* = 0.14), with zero heterogeneity (*I*^2^ = 0%). At 1-year follow-up, pooled analyses showed no significant differences in all-cause death (RR = 1.37, *P* = 0.62) or myocardial infarction (RR = 1.29, *P* = 0.56), also with consistent homogeneity (*I*^2^ = 0%).

**Conclusion:**

HCR demonstrates comparable safety and effectiveness to CABG for MVCAD, with similar rates of key outcomes at both 30 days and 1 year. Further research with larger samples and longer follow-up is recommended to confirm these findings.

## Introduction

Coronary artery disease (CAD) is characterized by insufficient blood and oxygen delivery to the myocardium. This is due to coronary artery occlusion and/or stenosis, which causes a mismatch in oxygen supply and demand. Most commonly, it involves the formation of plaques within the lumen of the coronary arteries, which inhibits blood flow. It is the main cause of death around the world. CAD was responsible for an estimated 2.2% of the overall global illness burden and 32.7% of cardiovascular diseases^[1,2]^. Previous research has demonstrated that cardiovascular diseases may remain clinically silent and are often detected only during autopsy in cases of sudden death, emphasizing the importance of early recognition and intervention to reduce mortality^[3,4]^. Diabetes mellitus, high blood pressure, smoking, hyperlipidemia, obesity, and psychological stress are among the risk factors^[5,6]^. Coronary artery bypass graft (CABG) and percutaneous coronary intervention (PCI) are surgical treatments of choice in CAD. Myocardial revascularization by CABG[7], is a major procedure in which occlusive atheromatous lesions in the arterial bed of a patient’s heart are bypassed with either an autologous vein or, less commonly, arteries. The bypass again ensures that blood supply is restored to the ischemic myocardium, thus regaining functionality, essential life, and relief from anginal pain[8]. The internal mammary artery exhibits the highest long-term patency (>90% at 10 years) and is spared from atherosclerosis in almost 96% of individuals[9]. It is associated with significantly reduced mortality, myocardial infarction (MI), and recurrent angina and possesses an outstanding long-term patency rate[10]. PCI, also referred to as percutaneous transluminal coronary angioplasty, is a less invasive therapeutic approach that seeks to restore Percutaneous Arterial Patency by relieving stenosis or occlusion of the coronary artery to ensure adequate tissue perfusion of ischemic tissues. This is normally done by several methods; the commonest being balloon angioplasty of the narrowed area or insertion of a stent to ensure that the artery remains open^[11,12]^. PCI with drug-eluting stents and CABG for the effective treatment of multivessel CAD has gained much attention in the recent past. This hybrid method is intended to achieve the best revascularization when not all targets can be grafted. By using this concept, complicated LITA to LAD grafting can be conducted with minimally invasive CABG techniques, and the entirety of further major stenosis will be solved by PCI[13].

**HIGHLIGHTS**

- Hybrid coronary revascularization (HCR), which combines coronary artery bypass grafting (CABG) and percutaneous coronary intervention, showed no significant difference from traditional CABG in terms of 30-day and 1-year outcomes, such as all-cause mortality, stroke, myocardial infarction, and need for blood transfusion.
- The risk ratios for all outcomes were statistically nonsignificant, with *P*-values >0.05, indicating similar safety and efficacy profiles.
- All included studies showed zero heterogeneity (*I*^2^ = 0%), suggesting consistent results across different trials and patient populations.
- The study recommends larger, high-quality randomized trials with longer follow-up to better evaluate long-term outcomes and validate HCR as a viable alternative to CABG.

We conducted this study to assess the long-term outcomes of HCR (combination of CABG and PCI) to conventional revascularization procedures since the results indicate that a hybrid strategy may be more advantageous.

## Methods

### Protocol and registration

The Preferred Reporting Items for Systematic Reviews and Meta-Analyses criteria and TITAN checklist were followed in the conduct of this systematic review and meta-analysis[14]. And detailed instructions from the Cochrane Handbook for Systematic Reviews of Interventions[15]. The study was registered in PROSPERO with registration ID CRD420251017408.

### Eligibility criteria

#### Population

Studies involving patients with confirmed angiography or multiple coronary artery disease. The main intervention is HCR, with the comparator group comprised of conventional revascularization using CABG or PCI. The primary outcomes of interest included short-term (30 days) cardiovascular events, including all causes of death, MI, stroke, and blood transfusion. In addition to 1-year events, including all causes of death and MI. We included only randomized controlled trials (RCTs). Non-RCT comparative trials and observational studies were excluded.

### Database search

A thorough investigation of the scientific literature was carried out by utilizing four prominent databases (PubMed, Cochrane Central Register of Controlled Trials (CENTRAL), Web of Science, and Scopus) to locate published publications using a specific search strategy until September 2024, with minor modifications made to accommodate the specific requirements of each database. Our Search considered research articles published in the English language only. Each database’s search method is available in Table 1.

**Table 1:** The search query of each database

Database	Search strategy	Records
PubMed	(“Hybrid Coronary Revascularization”[Title/Abstract] OR “HCR”[Title/Abstract] OR (“Hybrid”[Title/Abstract] AND “Coronary Revascularization”[Title/Abstract]) OR (“Coronary Artery Bypass”[Mesh] AND “Percutaneous Coronary Intervention”[Mesh])) AND (“Coronary Artery Bypass”[Mesh] OR “Percutaneous Coronary Intervention”[Mesh] OR “PCI”[Title/Abstract] OR “CABG”[Title/Abstract] OR “Revascularization”[Title/Abstract]) AND (“Long-Term Outcome”[Title/Abstract] OR “Long-Term Results”[Title/Abstract] OR “Follow-Up Studies”[Mesh] OR “Prognosis”[Mesh])	592
Scopus	(TITLE-ABS-KEY (“Hybrid Coronary Revascularization”) OR TITLE-ABS-KEY(“HCR”) OR (TITLE-ABS-KEY(“Hybrid”) AND TITLE-ABS-KEY (“Coronary Revascularization”))) AND (TITLE-ABS-KEY (“Coronary Artery Bypass”) OR TITLE-ABS-KEY (“Percutaneous Coronary Intervention”) OR TITLE-ABS-KEY(“PCI”) OR TITLE-ABS-KEY(“CABG”) OR TITLE-ABS-KEY(“Revascularization”)) AND (TITLE-ABS-KEY (“Long-Term Outcome”) OR TITLE-ABS-KEY (“Long-Term Results”) OR TITLE-ABS-KEY (“Follow-Up Studies”) OR TITLE-ABS-KEY(“Prognosis”))	744
Web of Science	(TS = (“Hybrid Coronary Revascularization”) OR TS = (“HCR”“) OR TS = (“Hybrid” AND “Coronary Revascularization”)) AND (TS = (“Coronary Artery Bypass”) OR TS = (“Percutaneous Coronary Intervention”) OR TS = (“PCI”) OR TS = (“CABG”) OR TS = (“Revascularization”)) AND (TS = (“Long-Term Outcome”) OR TS = (“Long-Term Results”) OR TS = (“Follow-Up Studies”) OR TS = (“Prognosis”))	546
Embase	(“hybrid coronary revascularization”:ti,ab,kw OR “HCR”:ti,ab,kw OR (“hybrid”:ti,ab,kw AND “coronary revascularization”:ti,ab,kw)) AND (“coronary artery bypass”:ti,ab,kw OR “percutaneous coronary intervention”:ti,ab,kw OR “PCI”:ti,ab,kw OR “CABG”:ti,ab,kw OR “revascularization”:ti,ab,kw) AND (“long term outcome”/exp OR “follow up”/exp OR “long term result”:ti,ab,kw OR “prognosis”/exp)”	381

### Study selection and data extraction

To eliminate any instances of duplication, the search results were imported into the EndNote software version 20[16]. Afterward, we assessed the titles and abstracts of the remaining papers using the Rayyan tool[17] to determine their suitability for the study. Two reviewers independently screened titles/abstracts and extracted data. Disagreements were resolved by discussion, and if necessary, a third reviewer acted as arbitrator. The articles that were selected for inclusion underwent a comprehensive evaluation process. Furthermore, we meticulously reviewed the citations used in the chosen publications to ensure a comprehensive exploration of potential directions for future research. In the end, two impartial evaluators manually collected the data and recorded the results on a standardized Google page. Extracted data included study characteristics: Author, year of publication, country, study design, and average follow-up period. Participant demographics included Sample size, age, male sex distribution, Previous condition, ejection fraction, SYNTAX score, and associated comorbidities, in addition to outcomes reported in the study comparing HCR to conventional therapy, and risk of bias assessment domains.

### Risk of bias assessment

We used the updated Cochrane risk-of-bias tool for randomized trials (ROB 2)[18] to evaluate the potential for bias in the clinical trials that were included. The tool’s organization is built on distinct bias domains, each of which deals with a certain facet of trial design, execution, and reporting. To collect data regarding trial characteristics relevant to potential bias, each domain uses a unique set of “signaling questions.” The answers to these questions are used by an algorithm to provide a suggested estimate of the probability of bias in each domain. This assessment has the potential to show “Some worries,” “Low,” or “High” risk of bias. Every domain was examined in turn by two different reviewers.

### Data synthesis and analysis

Review Manager software (RevMan v.5.4)^[19]^ was used to conduct a meta-analysis on the retrieved outcome data that were found in at least three of the included studies. The frequency of occurrences and the total number of patients were pooled as a risk ratio (RR) with a 95% confidence interval (CI) for dichotomous outcome data. A *P* < 0.05 threshold for statistical significance was established. Rather than using a fixed effect model, we choose to use a random effect model (inverse variance) to account for the heterogeneity of in-between studies and produce a more conservative effect estimate. The chi-square test was employed to assess heterogeneity, ensuring that an *I*^2^ value of greater than 40% and a significance level of *P* ≤ 0.1 were met. We place greater emphasis on the *I*^2^ value, as it performs better than the chi-square test when the number of included studies is small.

## Results

### Search results and study selection

**Figure 1.**
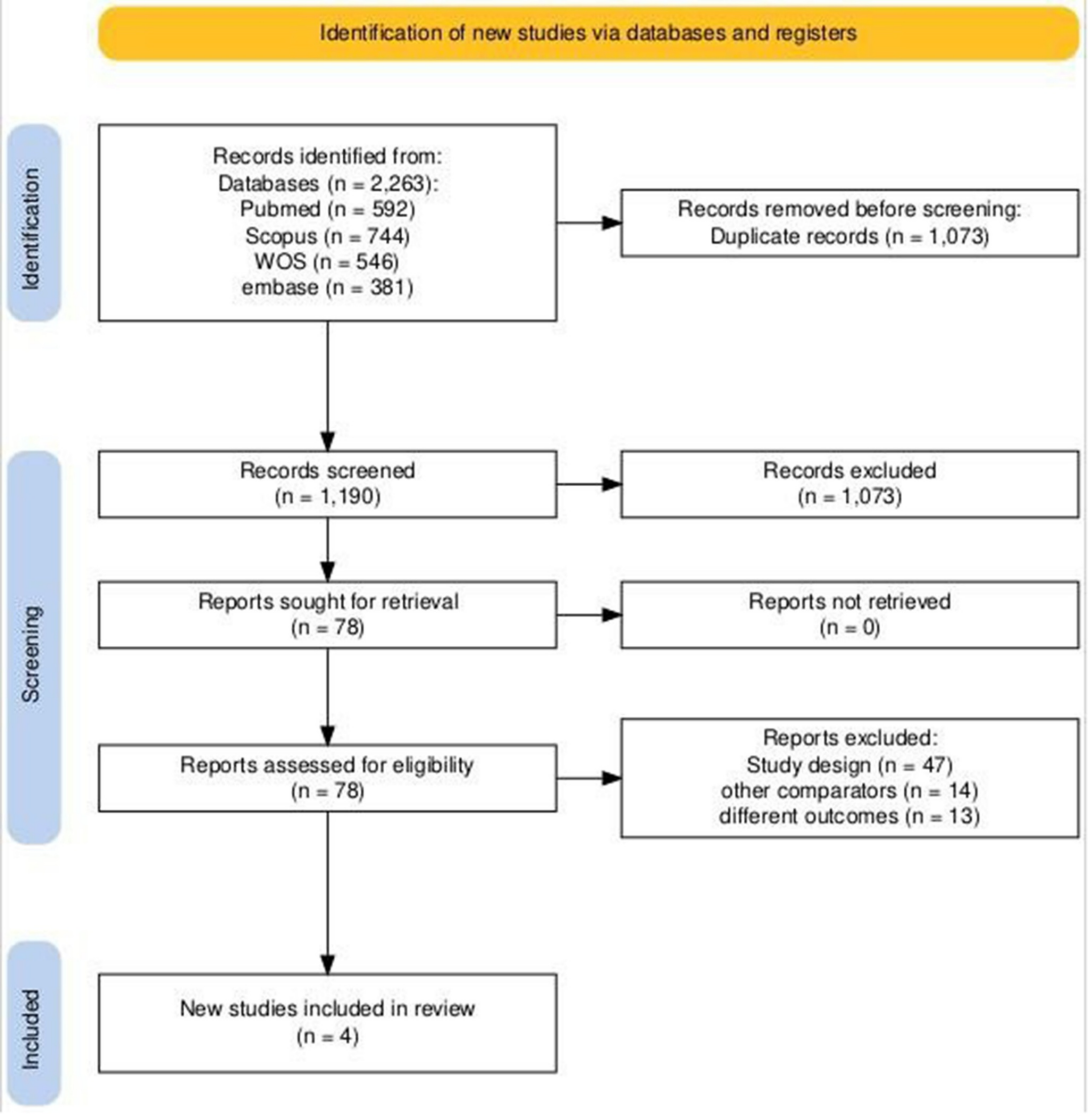
PRISMA flow diagram of the included studies.

After conducting a comprehensive analysis of the existing literature, we discovered 2263 relevant records across various databases such as PubMed, Embase, Scopus, and Web of Science. EndNote was used to optimize the data entry process, resulting in a final count of 1190 records after eliminating 1073 duplicates. After carefully examining the titles and abstracts, we chose 78 articles that met our study criteria. After carefully reviewing the entire text, we eliminated 74 records that did not meet specific criteria, leaving us with a final selection of four studies (Gasior 2014[10], Ganyukov 2020[20], Micovic 2014[21], and Esteves 2019[3]) that were included for qualitative synthesis and meta-analysis. Furthermore, the reference lists of these research studies were thoroughly scrutinized to identify any relevant publications that might have been missed initially. Figure 1 illustrates the search methodology and the number of papers that were included and discarded.

### Characteristics of included studies

The meta-analysis and systematic review included four studies. Studies with sample sizes ranging from 20 to 200 patients carried out worldwide and published in different years were included. Randomized clinical comparison trials involving individuals with various CADs comprised all of the included studies. All of the included trials contrasted one of the standard therapies – PCI or CABG – with hybrid coronary revascularization, which combines PCI with stent technology. Since there is only one trial showing results comparing HCR to PCI, a meta-analysis comparing HCR to CABG was carried out. The studies’ mean ages varied from 61.1 to 70 years. At baseline, there were no discernible differences between the groups. Table 2 shows a more detailed summary of included studies, and Table 3 shows baseline characteristics of patients in the included studies. Randomized clinical comparison trials involving individuals with stable, chronic multivessel CAD were included. Emergency or acute MI–related revascularization was not part of any included trial.

**Table 2:** The summary of the included studies

								Sample size	
Study ID	Study design	Registration number	Location	Population	Intervention	Comparator	Average follow-up	Intervention	Comparator
Gasior 2014	RCT	NCT01035567	Poland	Patients with multivessel coronary artery disease, including the left anterior descending artery, with a critical lesion (>70%) in at least one major epicardial vessel, are referred for conventional surgical revascularization	HCR (combination of minimally invasive direct coronary artery bypass and percutaneous coronary intervention with a stent)	CABG (coronary artery bypass grafting)/OPCAB (off-pump coronary artery bypass grafting)	12 months	98	102
Ganyukov 2020	RCT	NCT01699048	Russia	Patients with confirmed multivessel coronary artery disease with LAD and significant stenosis in a 2.5 mm diameter non-LAD epicardial vessel are amenable to PCI, CABG, and HCR	HCR (minimally invasive and robotic coronary artery bypass grafting + PCI)	G2: CABG, G3: PCI	12 ± 1 months	52	G2: 50, G3: 53
Micovic 2014	RCT		Serbia	Patients with severe triple-vessel coronary artery disease or significant left main stenosis, unsuitable for percutaneous treatment, and those with significant carotid artery disease manageable by surgery or percutaneous intervention, are eligible for this treatment	Hybrid carotid artery stenting (CAS) and CABG	Concomitant carotid eversion endarterectomy (CEA) and coronary bypass surgery	1 month	10	10
Esteves 2019	RCT	NCT02226900	Brazil	Patients with a triple-vessel coronary disease with a Syntax score of 22 or higher, suitable for hybrid treatment or CABG, aiming for complete revascularization in all cases	HCR (surgical grafting of the LIMA to the left anterior descending artery without the assistance of cardiopulmonary bypass plus PCI)	CABG (surgical grafting of the LIMA)	802 ± 500 days (26.73 + 16.67 months)	40	20

**Table 3:** The baseline characteristics of patients in the included studies

Study ID	Study arms	Sample size	Age mean (SD)	Sex (male), *n* (%)	BMI, mean (SD)	Ejection fraction (%), mean (SD)	SYNTAX score, mean (SD)	Previous condition, *n* (%)		Smoker, *n* (%)	Hypertension, *n* (%)
								MI	Stroke		
Gasior 2014	HCR	98	6.31 (8.2)	78 (79.6)	28.2 (3.3)	49.8 (6.3)	23.4 (6.3)	52 (53.1)	4 (4.1)	30 (30.6)	87 (88.8)
	CABG	102	63.9 (8.4)	73 (71.6)	29.1 (4.2)	50.7 (7)	22.8 (5.3)	59 (57.8)	5 (4.9)	36 (35.3)	81 (82.4)
Ganyukov 2020	HCR	52	62 (7.4)	39 (75)	NR	56.2 (6.3)	19.4 (3)	27 (51.9)	4 (7.7)	24 (46.1)	34 (65.4)
	CABG	50	61.3 (6.8)	35 (70)	NR	54 (7.4)	19.3 (3)	28 (56)	3 (6)	25 (50)	33 (66)
	PCI	53	61.7 (7.7)	37 (69.8)	NR	53.3 (9.9)	19.5 (2.7)	31 (58.5)	3 (5.7)	25 (47.2)	36 (67.9)
Micovic 2014	CAS/CABG	10	65 (7)	8 (80)	NR	44.3 (12.4)	NR	5 (50)	2 (20)	NR	9 (90)
	CEA/CABG	10	70 (7)	5 (50)	NR	43.4 (13.3)	NR	7 (70)	3 (30)	NR	8 (80)
Esteves 2019	HCR	40	61.1 (8)	30 (75)	28 (3.1)	59.8 (7.6)	28.3 (4.7)	NR	NR	12 (30)	NR
	CABG	20	61.1 (10)	18 (90)	27.7 (3.9)	61.3 (6.7)	29 (4.3)	NR	NR	2 (10)	NR

### Risk of bias

**Figure 2.**
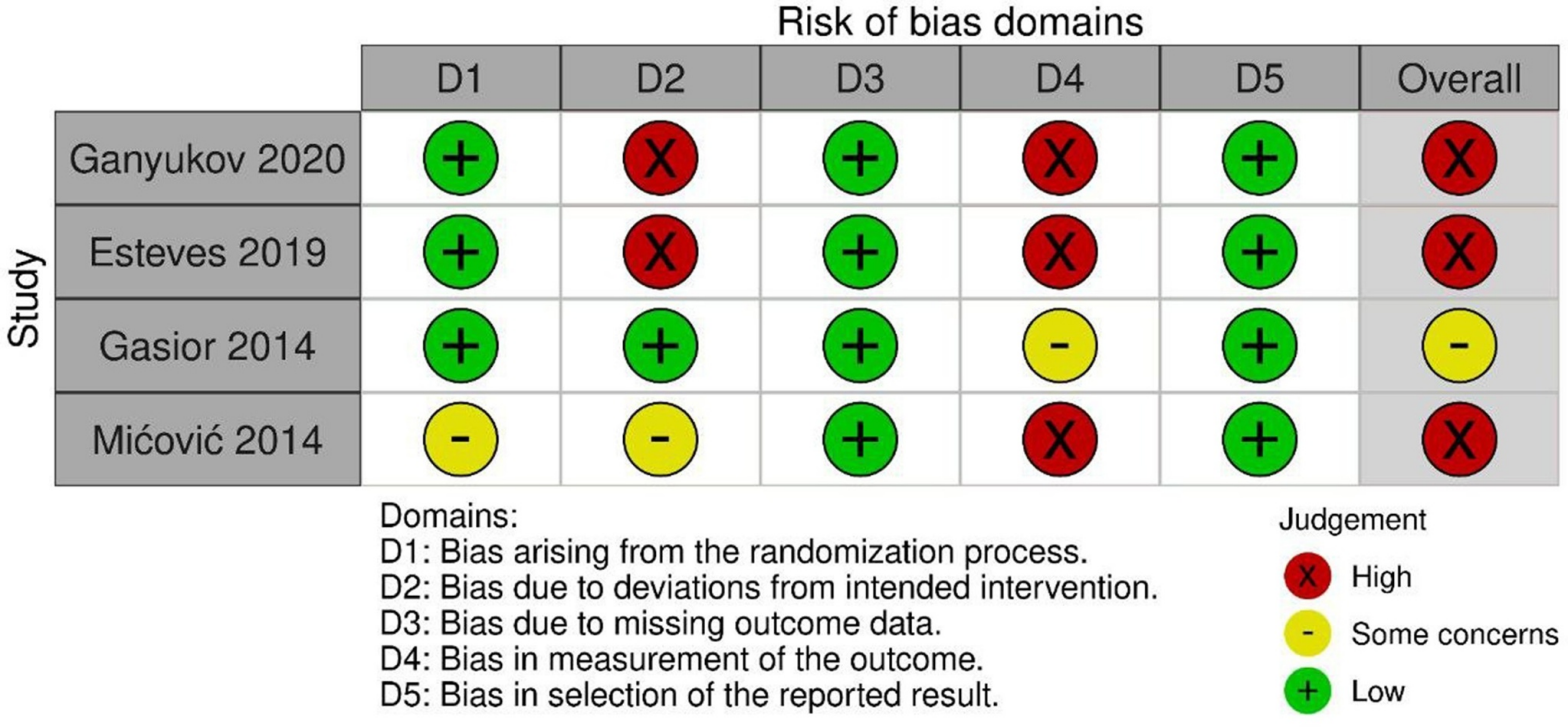
Risk of bias assessment graph for the included RCTs.

The assessment of potential bias within the included RCTs was conducted using the ROB2 tool. The risk of bias assessment revealed that three clinical trials (Ganyukov 2020, Esteves 2019, and Mićović 2014) were deemed as high risk of bias due to one or high-risk domains. Moreover, one clinical trial (Gasior 2014) showed some concerns about bias in the measurement of outcomes. Figure 2 shows the detailed risk of bias graph about the judgment of each domain in all included trials.

## 30-day outcomes

### 30-day all-cause death

**Figure 3.**
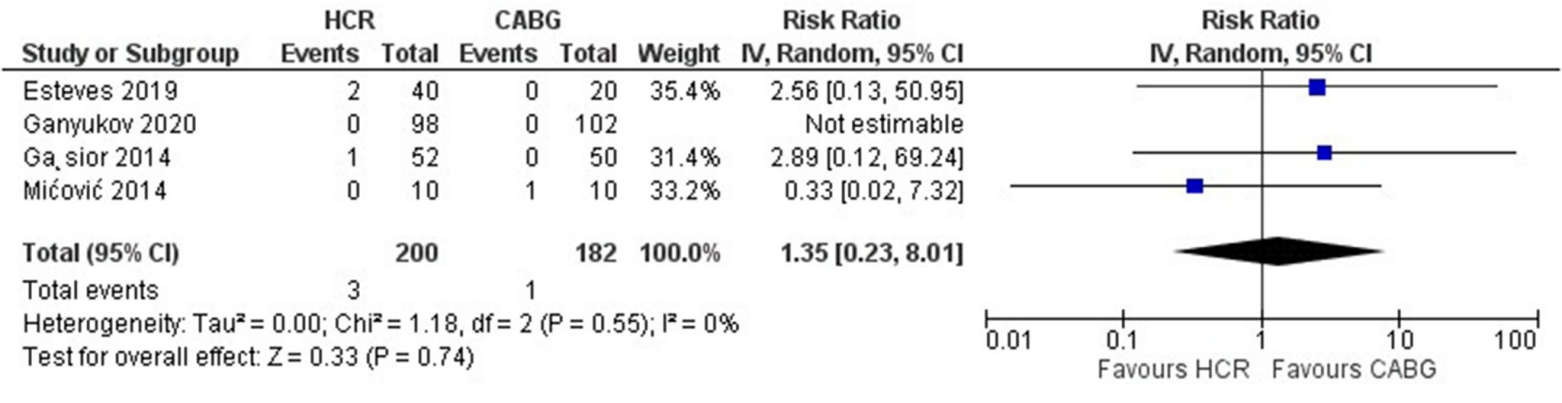
Forest plot of 30-day all-cause death analysis.

Data were extracted from four clinical trials with a total sample size of 382, and a meta-analysis was then conducted. The results of the meta-analysis showed no statistically significant difference between HCR and CABG regarding 30-day all-cause death (RR = 1.35, 95% CI [0.23, 8.01], *P* = 0.74), and pooled results were homogenous (*P* = 0.55; *I*^2^ = 0%), as shown in Figure 3.

#### Stroke

**Figure 4.**
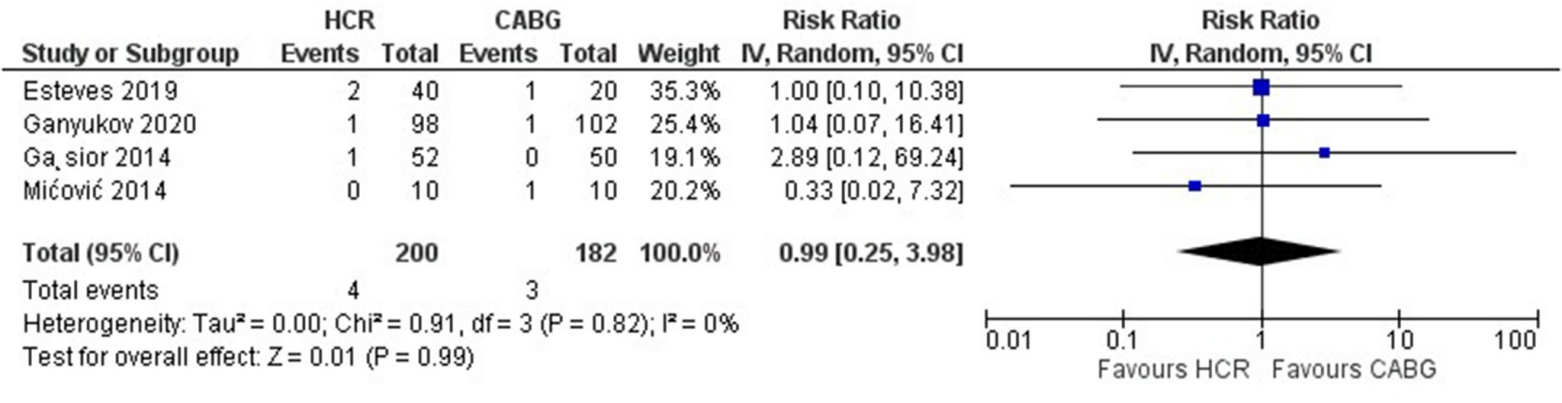
Forest plot of 30-day stroke analysis.

Results of the overall effect of 30 days of stroke occurrence showed no statistically significant difference between HCR and CABG (RR = 0.99, 95% CI [0.25, 3.98], *P* = 0.99) with no heterogeneity detected among studies as indicated by the Chi-squared *P*-value of 0.82 and *I*^2^ equals 0%. Figure 4 illustrates a forest plot of 30 days of stroke outcome.

#### Myocardial infarction

**Figure 5.**
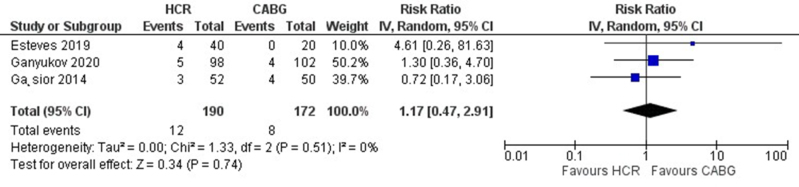
Forest plot of 30-day MI analysis.

In terms of the overall effect of 30-day MI, there was no statistically significant difference between the HCR group and CABG group (RR = 1.17, 95% CI [0.47, 2.91], *P* = 0.74); and pooled results were homogenous (*P* = 0.51, *I*^2^ = 0%) as illustrated in Figure 5.

#### Blood transfusion

**Figure 6.**
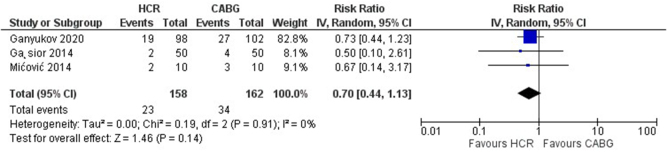
Forest plot of 30-day blood transfusion analysis.

The pooled effect of an incident of blood transfusion during the hospital stay or after discharge showed no statistically significant difference between both groups, HCR and CABG (RR = 0.70, 95% CI [0.44, 1.13], *P* = 0.14), with no heterogeneity detected among studies (*P* = 0.91, *I*^2^ = 0%) as shown in Figure 6.

### 1-year assessed outcomes

#### All-cause death

**Figure 7.**
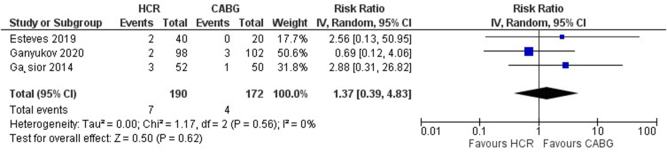
Forest plot of 1-year all-cause death analysis.

With a total sample size of 362 patients’ data extracted from three clinical trials, the pooled treatment effect showed no statistically significant difference between HCR and CABG (RR = 1.37, 95% CI [0.39, 4.83], *P* = 0.62) with no heterogeneity detected (*P* = 0.56; *I*^2^ = 0%) as illustrated in Figure 7. Micovic 2014 was excluded from 1-year analyses due to absence of long-term follow-up data.

#### Myocardial infarction

**Figure 8.**
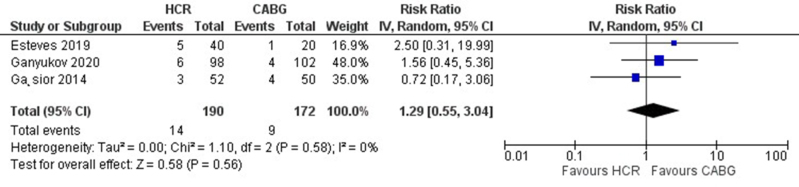
Forest plot of 1-year MI analysis.

Results of the meta-analysis showed no statistically significant difference between HCR and CABG (RR = 1.29, 95% CI [0.55, 3.04], *P* = 0.56) with no heterogeneity detected (Heterogeneity: Tau^2^ = 0.00; Chi^2^ = 1.10, df = 2, *P* = 0.58; *I*^2^ = 0%) as shown in Figure 8.

##### Certainty of evidence

Formal GRADE analysis was not feasible due to the limited studies. However, given the high risk of bias in three trials and small sample sizes, the certainty of evidence is considered low.

## Discussion

Hybrid coronary revascularization, or HCR, blends the principles of” off-block” surgical technique and PCI, thus being an alternative to standardized CABG[4]. Although HCR appears to lessen one’s surgical stress and time of convalescence, studies about it do compare it with standard CABG and PCI, and none are doing so convincingly^[22,23]^. There are both positive and negative results concerning its effectiveness and safety measures, thus, there is a need to look for all aspects in the available literature. Thus, this systematic review and meta-analysis were designed to evaluate the outcomes of HCR against the traditional methods of treating CAD, with a clear outcome of the effectiveness and safety profile of the HCR procedure.

## Main findings

The results of this systematic review and meta-analysis provide valuable information regarding the effectiveness and safety of HCR in traditional CABG. Addressing the first short-term outcomes, defined as any complication occurring within 30 days after the performance of the procedure, HCR and CABG did not reveal any differences for the overall mortality, despite being all-cause. This corresponds to the fact that in groups undergoing different surgical interventions, the difference in the risk of death within the early postoperative period remains insignificant.

However, it is noteworthy that, when analyzing the occurrence of the stroke within the same 30-day period, there was no evidence for effectiveness differential in favor of CABG in comparison with HCR. This is important since stroke is one of the most severe complications that may arise following cardiovascular procedures. The fact that the HCR group did not experience an increased risk of stroke is an indication that the hybrid approach is almost as safe as the conventional CABG method.

As for MI, the other major outcome, this meta-analysis demonstrated that there was no difference in 30-day re-MI rates between HCR and CABG. This suggests that the hybrid technique does not increase the risk of perioperative MI, which is one of the concerns of patients suffering from CAD. Meaning that the MI post-procedure rates were approximately equal, in that HCR is as safe and effective as CABG in averting early postoperative MIs.

In this analysis, attention was also paid to the necessity of blood transfusion during or after the hospital stay as one of the determinants of the invasiveness of the procedure, as well as the recovery of the patient. The findings were that there was no difference in this aspect between HCR and CABG, proving that lower invasive HCR does not necessarily mean less blood transfusion requirements. This finding suggests that such blood loss and postoperative anemia management, regardless of the modes of operation, produces the same amount of blood loss and postoperative anemia management by the practitioners.

The analysis of 1-year outcomes revealed that there was no considerable variation in terms of all-cause mortality as well as MI rates between HCR and CABG. Studies suggest that HCR may offer a better quality of life and shorter recovery time. However, due to limited long-term RCTs, these benefits remain inconclusive. Future trials should focus on patient-centered outcomes.

The consistency of long-term outcomes across key indicators reinforces the earlier conclusion that less invasive strategies, such as HCR, may offer comparable efficacy and safety to conventional CABG. This finding is especially relevant for patients who prioritize factors like long-term health, faster recovery, and improved quality of life when choosing between treatment options. Although revascularization rates, bleeding events, renal failure, and patient-reported quality of life are relevant outcomes, limited data precluded their inclusion. These remain important areas for future trials. Cardiac rehabilitation plays a vital role in CAD by improving recovery, reducing recurrent events, and enhancing long-term outcomes^[22]^.

These results are consistent across temporal boundaries (30 days and increased), underscoring the capacity HCR has in improving the quality of care in the management of CABG, especially in patients who are reluctant to undertake highly invasive surgeries because of prolonged recovery periods.

In interpreting these findings, it is important to consider the methodological quality of the included RCTs. According to the ROB2 assessment, three of the four trials were judged to have a high risk of bias in at least one domain, and sample sizes across studies were small. These limitations reduce the overall certainty of evidence and suggest that the pooled estimates should be interpreted with caution. Larger, methodologically robust trials are needed to strengthen confidence in these conclusions.

### Strengths and limitations

This meta-analysis report has one major strength, which is the homogeneity observed within the studies that have been covered in the meta-analysis, as is indicated by the constant lack of heterogeneity in the pooled results. It follows from this homogeneity that these outcomes are plausible and are likely to be applicable in a variety of clinical contexts and patient cohorts. Further, the arguments contained in the present review are based on the data derived from the RCTs, which are regarded as the most reliable type of clinical research. Conducting RCTs is beneficial so that there is no shifting of the outcome when conducting the clinical trials, because this increases the level of confidence in the deductions made. Only English-language studies were included, which may introduce language bias. Notably, there were some limitations that should be considered when interpreting the findings. First, our results are based on a limited number of RCTs (4), and they were of a small sample size. So, further high-quality RCTs with larger sample sizes are needed to validate the results in the future. Also, with small sample sizes, the meta-analysis may be underpowered to detect modest differences, increasing the risk of type II error. Variability in surgical approaches (robotic vs minimally invasive vs open HCR) and PCI techniques likely contributes to clinical heterogeneity and limits generalizability. Additionally, studies reporting other clinical outcomes, such as revascularization, major bleeding, renal failure, and residual myocardial ischemia, were not enough to perform a pooled analysis. Micovic 2014 was included because it was an RCT categorized as “hybrid revascularization”; however, as it involved hybrid carotid stenting plus CABG rather than direct HCR vs CABG in coronary disease, its clinical relevance is limited. Although there was no heterogeneity (*I*^2^ = 0), variations in the sample size, materials used in the operation, operating surgeons’ technique, and postoperative regimen could introduce biases and potentially impact the pooled outcomes. However, these variations give the study results high generalizability and external validity. No analysis beyond 2 years was possible. High-risk subgroups (diabetics, elderly) could not be analyzed due to data scarcity.

### Research recommendation

Future trials should systematically assess patient-centered outcomes, including quality of life, recovery time, and long-term graft/stent patency, which remain critical gaps in the literature. Even though the findings for the 2-year follow-up are available, we did not evaluate them because they were only found in one study. Therefore, longer-term studies are needed to ascertain whether or not there will be a substantial difference between HCR and CABG. Furthermore, it is imperative to carry out comparative analyses with PCI. Finally, because there were so few papers, we were unable to assess the robustness of the results using publication bias or leave-one-out analysis.

## Conclusion

HCR could be an alternative to a CABG, especially in patients who are most likely to benefit from the procedure with less invasive measures and satisfactory clinical results. Current evidence suggests HCR may have comparable short-term outcomes to CABG, but certainty is low due to small sample sizes and high risk of bias in most trials. According to the analysis, there was no statistical difference between the two approaches concerning patients’ all-cause mortality, incidence of stroke, MI, or revascularization, as well as the need for blood transfusion. These results are extended based on the findings’ universality from the examined research involving these patients. However, to ascertain the validity of these results and any potential variations about the longer-term effects of these two treatment modalities, larger-scale, longer-term studies are acknowledged as being necessary.

## Data Availability

The data that constitute the findings of the study are available in the study itself.

## Research registration unique identifying number (UIN)

PROSPERO: CRD420251017408.
